# Effects of Vestibular Damage on the Sleep and Expression Level of Orexin in the Hypothalamus of Rats and Its Correlation with Autophagy and Akt Tumor Signal Pathway

**DOI:** 10.1155/2022/2514555

**Published:** 2022-06-26

**Authors:** Gangli Yan, Fengguang Li, Zhiwei Tao, Xiaobing Xing, Ziying Zhou, Xijia Wang, Jinxia Zhou

**Affiliations:** ^1^Department of Neurology, Puren Hospital Affiliated to Wuhan University of Science and Technology, Wuhan, Hubei 430081, China; ^2^Department of Neurology, Wuhan Asia General Hospital, Wuhan, Hubei 430090, China

## Abstract

The aim of this study was to investigate the effect of vestibular disruption on autophagy-related proteins and the tumour-associated pathway P13K/Akt in rat sleep and its hypothalamus tissue and to examine whether catechins trigger tumour autophagy. Healthy adult male rats were randomly selected and divided into the vestibular damage group, the sham operation group, and the control group, with 8 rats in each group. A vestibular damage model was established through penetrating the tympanic membrane of the external auditory canal by injecting sodium p-aminophenylarsonate. The electroencephalogram (EGG) activity was used to record the sleep-wakefulness cycle of rats, and the expression levels of hypothalamic orexin (orexin) mRNA and autophagy proteins were detected. Primary hippocampal neurons were intervened with orexin at different concentrations and at different times to detect cell viability and the expression of autophagy protein and P13K/Akt signal pathway protein. The results showed that compared with the control group and the sham operation group, NREM duration in the vestibular damage group decreased significantly (*P* < 0.05), while its  W  time increased significantly (*P* < 0.05). The expression level of orexin mRNA in the hypothalamus of the vestibular damage group was significantly higher than that of the other two groups (*P* < 0.05), the expression of autophagy microtubule-related proteins LC3B and Beclin-1 increased significantly (*P* < 0.05), and the protein expression level of p62 decreased significantly (*P* < 0.05). After orexin intervention, compared with the control group, the expression of Beclin-1 protein that positively correlated with autophagy decreased significantly (*P* < 0.05) and the expression of mTOR, PDK1, and Akt protein increased significantly (*P* < 0.05). Compared with the orexin intervention group, the expression of Beclin-1 and LC3B proteins in cells of the orexin receptor inhibitor (Almorexant) group, the autophagy activator (Rapamycin) group, the orexin + Almorexant group, and the orexin + Rapamycin group increased significantly (*P* < 0.05), and the expression of mTOR, PDK1, and Akt proteins decreased significantly (*P* < 0.05). Catechins trigger autophagy in part by regulating the p-Akt/p-mTOR and P13K pathways and by stimulating the MAPK pathway. Catechins initiate apoptosis in common tumour types of hepatocellular carcinoma cells by activating autophagy-related pathways. The conclusion is that vestibular damage can affect the sleep-wakefulness cycle of rats; the level of autophagy in hypothalamic tissue is upregulated and may affect cell proliferation and activity through mTOR-P13K/Akt, which has a certain reference value for tumor formation and provides a basis for the research of insomnia or sleep disorders caused by tumors. Autophagy activation is a key process by which catechins promote apoptosis in tumour cells, providing an avenue for more research on the use of catechins-rich diets for cardiovascular protection in the treatment of tumours.

## 1. Introduction

Sleep is the most essential physiological function of human body, which is both important and complex. It involves many parts of the central system and is affected by many factors. After years of research, its mechanism is still not yet very clear to us, so there exist many hypotheses to date. Modern medicine believes that the disorder of the sleep-wakefulness cycle can lead to the occurrence of insomnia, and its pathogenesis is closely related to the sleep-wakefulness cycle [1]. Modern studies have found that insomnia is caused by the dysfunction of systems (brainstem raphe nucleus, dorsal thalamus, hypothalamus, suprachiasmatic nucleus, and cerebral cortex) related to brain sleep and the imbalance of sleep structure and process resulted by the disorder of sleep factors (SFs) [2]. The vestibular system is located in the inner ear and includes peripheral vestibular organs and vestibular neurons. It mainly receives motion and gravity information [3], which is transmitted to the vestibular nucleus (VN) of the brain stem through vestibular organs, and then further transmitted to other brain regions after integration. It participates in a variety of adjustment processes, such as respiration, blood pressure [4, 5], circadian rhythm of central body temperature [6], and blood redistribution during movement and posture change [7, 8]. These physiological adjustments are closely related to sleep.

Orexin is a small-molecule neuropeptide [9, 10], which is mainly synthesized and secreted by the lateral hypothalamus. Orexin is expressed not only in hypothalamus, but also in gastrointestinal tract, kidney, and pituitary. Orexin includes orexin A and orexin B [11, 12]. With the progress of research in recent years, orexin has been found to not only participate in food intake, but also affect sleep cycle, learning and memory, and drug addiction. Recently, the effect of hypothalamic arousal promoting neurotransmitter orexin on sleep and arousal is now a research hotspot and has attracted more and more attention. The lateral hypothalamic area is the main area of arousal initiation area, and orexin neurons exist exactly in this area. The arousal initiation system of orexin neurons in the posterolateral hypothalamic area controls the coordination and unity of the activities of each arousal regulatory system and plays a role in stimulating the cortex and maintaining arousal. The experimental results show that the drugs acting there can effectively make animals or humans sleep [13]. Autophagy is a defense mechanism and adaptive response to exogenous stimuli, which plays an important role in maintaining the synthesis and degradation balance of intracellular substances and participating in the orderly reuse of cellular components. At present, studies have found that orexin can activate mTORC1 in rat hepatocytes and mTORC1 of the HEK-293T cell line [14, 15], and mTOR-dependent pathway is a key signal molecule for autophagy regulation. Recent studies have found that chronic sleep deprivation causes abnormalities in the autophagy activity of cortex and hippocampal neurons [16].

In recent years, studies have found that with the gradual increase in the number of cancer patients, there is a very close relationship between sleep disorders and tumors. Some foreign research reports indicate that the prevalence of sleep disorders spontaneously reported in cancer populations is 30%–75% [17, 18]. The autophagy protein Beclin-1 plays an important role in the process of cell differentiation, apoptosis, and autophagy. It can participate in the occurrence and development of tumors by influencing the life cycle of tumor cells, regulating tumor cell apoptosis, and controlling tumor angiogenesis [19–22]. At present, Beclin-1 is generally considered as a tumor suppressor gene, and the deletion of its allele can be detected in a variety of tumors [23], while some studies have found that Beclin-1 can promote occurrence and development of tumors [24, 25]. However, there are relatively few studies on the effect of orexin on autophagy in the hypothalamus and the signal pathways related to tumors.

This study is mainly to establish a model for animals with vestibular damages to explore the effect of vestibular damage on the sleep of rats, as well as the effect of orexin expression and autophagy on hypothalamic tissues. It aims to further use orexin to stimulate hypothalamic neuron cells to detect autophagy, the expression of phage protein, and the possible mechanism of tumor-related regulation, which provides a basis for the study of insomnia or sleep disorders caused by tumors. Detection of expression of autophagy-related proteins and pathways with catechin stimulation on common tumour cells is of greater use with catechin-rich diets for the treatment of tumours and tumour-induced sleep disorders.

## 2. Related Works

### 2.1. Experimental Animal

The experimental animals were all adult male SD rats (provided by Animal Room of Wuhan University of Science and Technology), weighing 230–250 g. The animals were raised in cages and in a standard experimental environment (light for 12 h, temperature 22 ± 2°C, and humidity 50%) and could eat food and drink water freely. All animal experiments met the ethical requirements of experimental animals and were approved by the ethics committee.

### 2.2. Experimental Grouping and Model Preparation

Twenty-four rats were randomly divided into the vestibular damage group, the sham operation group, and the control group, with 8 rats in each group. In the vestibular damage group, rats were anesthetized by intraperitoneal injection of pentobarbital sodium (3 ml/kg), lying on their sides on a constant-temperature table for heat preservation, and injected with sodium p-aminophenylarsonate (100 mg/ml dissolved in 0.3 M sodium carbonate) by penetrating the tympanic membrane through the external auditory canal. The injection volume of each side was 0.1 ml, stuffed with an absorption sponge, and the drug absorption was promoted in the supine position. In the sham operation group, rats were anesthetized by intraperitoneal injection of pentobarbital sodium (3 ml/kg), lying on their side on a constant-temperature table for heat preservation. They were injected 0.1 ml sodium carbonate by penetrating the tympanic membrane through the external auditory canal, stuffed with an absorption sponge, and lying on their back to promote drug absorption. In the control group, rats were anesthetized by intraperitoneal injection of pentobarbital sodium (3 ml/kg) without any treatment.

### 2.3. Experiment 1: Changes of Vestibular Sleep-Wakefulness Cycle in Rats with Vestibular Damage

#### 2.3.1. Surgical Operation Recorded by EEG

Disinfect EEG electrodes with alcohol for standby. The rats were anesthetized by intraperitoneal injection of 10% chloral hydrate (0.35 ml/100 mg), the heads of rats were fixed with the ear pounds, and the ear pounds were fixed on the stereotactic instrument. The cranial top skin of rats was disinfected with Iodophor. After being shaved, the skin on the skull surface was cut off, and the surface tissue was removed. The front and rear fontanels were fully exposed to stop bleeding completely to ensure that the skull surface was dry. The electrode positioning position is as follows: the intersection of 1 mm in front of the coronal suture and 1 mm on the right side of the skull midline, and the intersection of 1 mm in front of the herringbone suture and 1 mm on both sides of the skull midline. Fix the EEG electrode at these three points, subject to no damage to the dura mater; EMG electrodes were inserted into the left superficial muscle layer and the right deep muscle layer of the rat neck, respectively. EEG and EMG electrodes were fixed on the surface of skull with dental cement. After waking up, the rats were fed in a single cage where they could eat and drink freely. One week after operation, EEG and EMG were recorded.

#### 2.3.2. EEG Activity Recording

Place the transparent plexiglass cylinder EEG recording device in the shielding cabinet, and put the rats into the EEG recording device to adapt to 1-2D. They can eat and drink freely and move freely. The photoperiod was 12 h with light and 12 h without light. The photoperiod was controlled by an automatic timer, and the light on and off times were 7:00 and 19:00, respectively. A rotating body was installed above the cylinder to ensure that the rats could move freely. One end of the wire was connected to the electrode of the rat head, and the other end was connected to the rotating body, which was then connected to the signal amplifier. The amplifier amplified and filtered the EEG signal (band-pass filtering between 0.3 and 30.0 Hz) and EMG signal (band-pass filtering between 100 and 300 Hz), and then directly input it into the computer through the analog-to-digital converter. The analog-to-digital conversion and data recording were controlled by SLEEPWAVE software. Each rat was recorded continuously for 24 hours (from 19:00 on the day to 19:00 on the next day), and data analysis was performed by the SleepSign software after the recording. According to the mode of manual analysis on the sleep of rats, the software was automatically analyzed and then manually corrected. Taking 10 s as the basic unit, the sleep-wakefulness cycle was divided into (1) nonrapid eye movement sleep period (NREMS): high amplitude and slow wave, EMG activity decreased significantly; (2) rapid eye movement sleep period (REM): characterized by low amplitude and continuous theta wave (6–10 Hz), no obvious muscle activity, and occasional muscle twitch; and (3) wake period (W): frontal occipital lobe leads out EEG with low amplitude and fast wave and obvious EMG signals. After correction, SleepSign software was used to count the sleep phase time of the rats within 24 hours. Total sleep time (TST) is the sum of the NREM time and the REM time. When two states (e.g., NREM and W) occurred simultaneously in a 10 s division cycle, the main part occupying 2/3 was defined as sleep state or arousal state.

### 2.4. Orexin mRNA Experiment 2: Effect of Vestibular Damage on Orexin mRNA and the Autophagy Level in Hypothalamus

#### 2.4.1. Orexin mRNA Effect of Vestibular Damage on Orexin mRNA

Three rats in each of the three groups were randomly selected. After weighing, they were intraperitoneally injected with anesthetic. After complete anesthesia, we cut open their abdominal cavities, intubated from the left ventricle, cut open the right atrial appendage, and let the normal saline flow out of the right atrium after peripheral circulation until the blood was completely flushed. Then, they were perfused and fixed with 0.1 mol/L phosphate buffer containing 4% paraformaldehyde (PBS, pH 7.4, 4°C). Until the bodies of rats were completely fixed, the judgment standard then was the rigidity of the rat tail. Take out the hypothalamic tissue, put it into a container containing liquid nitrogen for grinding, transfer the tissue powder to the EP tube containing 1 ml Trizol, mix it according to the volume ratio of no more than 1:10, and then mix it evenly and let it stand for 5 minutes. Add 200 ul of chloroform to the mixture and mix well. Centrifuge at 4°C with a speed of 12000 rpm, transfer the supernatant after 10 minutes, add 500 *μ*l of isopropanol to the supernatant, and mix evenly. After standing for 10 minutes, centrifuge again at 4°C with a speed of 12000 rpm for 10 minutes. Add 1 ml of 75% ethanol to the precipitate after removing the supernatant, shake with an oscillator for 10–15 seconds to make them fully mixed, and repeat centrifugation twice. After removing the supernatant again, open the cover and dry it in a 37°C incubator for 10 minutes, and add 30 *μ*l of nuclease-free H2O into the EP tube to dissolve the extracted RNA. RNA reverse transcription: use SYBR® Green I Kit (Takara Bio), gradually add required samples and reagents in proportion, namely, 5 × Primescript RT Master Mix, RNA samples, and RNA nuclease-free H2O. Mix them evenly and put them into the machine to run the program for reaction. At last, use the SYBR® Premix Ex Taq II kit (Takara Bio) to gradually add the required samples and reagents in proportion to SYBR® Premix Ex Taq II, ROX Reference Dye or Dye II, cDNA reaction solution, upstream primers, downstream primers, and distilled water. Use GAPDH as the internal reference gene for calibration and calculate the relative content of target gene expression by applying the 2^−ΔΔCt^ method.

### 2.5. Western Blot Detecting the Expression of Autophagy Protein in Rat Hypothalamus with Western Blot

Randomly select 5 rats from each group, cut their heads and take out the brains immediately, place them on crushed ice to separate the hypothalamic tissues on both sides, mark the remaining tissues and the left and right sides respectively, and place them in a −80°C box for later use. The expression of autophagy-related proteins in rat hypothalamus is then detected with Western blot.

The steps of Western blot are as follows:

  Protexin extraction: rinse the sample cells with PBS for 2 to 3 times, and add an appropriate volume of lyase mixed with protease inhibitor into the culture dish for 3 to 5 minutes. Shake the culture dish repeatedly to make the lysate contact with the cells more fully. After the cells are scraped from the culture plate, use a pipette to transfer them to a pre-prepared centrifuge tube (preferably done on ice). After 30 minutes of ice bath, blow the pipette repeatedly to prevent cells from being fully lysed and affecting the content of protein. After centrifugation at 12000 rpm for 5 minutes, the upper cool liquid in the test tube obtained is the total protein solution required for the experiment. Reserve 2 ul of BCA for quantification, and freeze the remaining protein at −80°C.
  BCA method for the determination of protein concentration: Prepare 25 mg/ml of protein standard solution in advance in strict accordance with the requirements of the protein concentration determination kit, and dilute part of it to 0.5 mg/ml. According to the number of samples, mix reagent A and reagent B in 50 : 1, and place them indoors for 24 hours to stabilize them. After carefully adding the sample to the 96-well plate according to the instructions, add 200 ul of working solution to each well and incubate it in an incubator at 37°C for 30 minutes. Put the sample plate into the microplate reader to measure the wavelength value of A562, and get the standard curve. After that, calculate the protein concentration in the experiment.
  SDS-PAGE electrophoresis: Prepare the glue according to the experimental arrangement. The upper glue (separation glue) is prepared to 15% and the lower glue (separation glue) is prepared to 5%. Add TEMED, shake it evenly, fill the glue water in it, and fill the remaining space with concentrated glue. After it, insert the comb. After it has solidified, add the samples and the markers. Add electrophoresis solution at the voltage of 90 V until the marker band appears. Then, adjust the voltage to 120 V until the bromophenol blue band shows up.
  Transfer the membrane under the condition of 4°C; set the current at 300 mA and the time at 30 minutes. After the membrane transfer is completed, cut the internal reference strips and the target strips required for the experiment.
  Sealing: place the transformed PVDF membrane in the configured 5% skimmed milk at room temperature, and use the experimental table concentrator to maintain the light shaking state. The sealing time is about 1 h.
  Immune reaction: primary antibody incubation: after removing the blocking solution, add diluted primary antibody, shake the table concentrator, and leave it overnight at 4°C. At room temperature, wash the TBST solution three times with a decolorizing table concentrator. After the secondary antibody required for the experiment is diluted, incubate the antibody for 30 minutes and wash it with PBS solution.
  Color development: prepare color development solutions ECL A and B according to the instructions. After color development, observe the experimental results according to the exposure time and exposure area, and compare the target bands of LC3B, Beclin-1, and use the optical density values of the target bands repressed by LC3B, Beclin-1, and p62 and the internal reference *β*-actin band to represent the relative expression value of each product.

### 2.6. Experiment 3: Effect of Orexin on the Autophagy Level and Akt Signal Pathway of Hypothalamic Neurons

#### 2.6.1. Culture of Primary Hypothalamic Neurons

After the newborn SD rats were anesthetized at low temperature on ice, clean and disinfect them with alcohol to separate the brain. Completely take out the brain tissue under the environment of sterile ultra clean table and place it in the anatomical liquid on ice. Carefully dissect the meninges under the microscope, blunt dissection, carefully take out the hypothalamus on both sides, put the tissue into the culture dish, and use the tissue scissors to cut the tissue into small pieces sized less than 1 mm^3^ as possible as we can. Put the chopped hypothalamic tissue into the digestive solution with DMEM: 0.25% trypsin = 1 : 1, digest at 37°C for 15 minutes (mildly shake it during the period to make the digestion more complete), and then add 20% DMEM mixture (including 10% FBS) to terminate the digestion. Transfer the obtained mixed solution from the culture dish to a 15 ml centrifuge tube, continue to add 20% DMEM mixed solution to about 6–7 ml, blow with a straw for about 6 minutes, suck out the supernatant after standing, then transfer it to a 200-mesh nylon net, and filter and collect the filtered solution into the centrifuge tube. Blow the pipette repeatedly and centrifuge the cell suspension at 850 rpm for 5 minutes. Discard the supernatant, add the cell precipitation into the 20% DMEM mixture, and then gently blow evenly to obtain the cell suspension. After counting the cells with a counter, inoculate them into a 6-well plate culture dish coated with polylysine in advance, and add about 2 ml of culture medium to each well. Place the 6-well plate in an incubator at 37°C, 5% CO2, and 85% humidity for cell culture. After 24 hours, extract out 20% DMEM culture medium and replace it with neurobasal neuron culture medium (2% B27, 0.2% glutamine). During the cell culture period, semiquantitatively change the medium for 2-3 days. After culturing for about 6–8 days, use the relevant drugs for intervention treatment to collect adherent cells and proceed to the next step.

#### 2.6.2. Orexin Effect of Orexin at Different Concentrations on the Viability of Hypothalamic Neurons

Configure orexin into 4 different concentrations of treatment solution of 0 mol/L, 10–11 mol/L, 10–9 mol/L, and 10–7 mol/L, and divide the hypothalamic neuron cells cultured in the same batch randomly into 4 groups. Use 4 different concentrations of orexin solutions to intervene in 4 groups of hypothalamic neuronal cells, then add about 2 ml of culture medium to each well to maintain the consistency of the number of cells per well, and place the well plate in an incubator for culture. Select six duplicate samples for each treatment. After 2 hours, detect the viability of hypothalamic cells with CCK-8 and select the optimal orexin intervention concentration.

#### 2.6.3. Orexin Effect of Orexin Intervention at Different Times on the Viability of Hypothalamic Neurons

Randomly divide the hypothalamic neurons cultured in the same batch into five groups. According to the optimal orexin intervention concentration selected in the previous step, intervene in 5 groups of 0 h, 2 h, 4 h, 6 h, and 8 h, respectively, and make relevant marks (time, intervention conditions). Then, add about 2 ml of culture medium to each well to maintain the consistency of the number of cells per well, and place the well plate in the incubator for culture. Select six duplicate samples for each experiment. Detect the viability of hypothalamic cells at different time with CCK-8.

#### 2.6.4. Orexin Akt Effects of Orexin on the Autophagy Level of Hypothalamic Neurons and Akt Signal Pathway

Divide the hypothalamic neurons cultured in the same batch randomly into 6 groups: (1) the control group, (2) the orexin group, (3) the orexin receptor blocker-Almorexant group, (4) the autophagy activator-rapamycin group, (5) the orexin + almorexant group, and (6) the orexin + rapamycin group. Orexin intervention concentration and intervention time are the best intervention concentration and intervention time selected according to the test results of the first two steps. According to the grouping design of this experiment, the proteins were extracted after the recovery of hypothalamic neurons in each group after intervention. Detect the expressions of autophagy-related proteins LC3B, Beclin-1, and p62, and PI3K/Akt-mTOR signal pathway mTOR, PDK1, and Akt with Western blot.

#### 2.6.5. Effect of Catechins on the Signalling Pathway at the Level of Autophagy in HepG2 Cells

HepG2 cells were treated with catechin gallate (EGCG) at intervention concentrations (0, 5, 25, and 125 mol/L) and labeled (time, intervention conditions), then about 2 ml of culture medium was added to each well to keep the number of cells in each well consistent, and the plates were incubated in an incubator. The effect of EGCG on the expression of Akt/mTOR signaling pathway-related proteins was detected by Western blotting.

#### 2.6.6. Data Statistics

Use SPSS24 software to conduct one-way analysis of variance (one-way ANOVA) on the test data and Duncan's test for multiple comparisons. Significant differences in test results were expressed as *P* < 0.05, and the results were expressed in the form of mean ± standard deviation (Table 1).

## 3. Results

### 3.1. Effect on the Vestibular Sleep-Wakefulness Cycle Changes in Rats with Vestibular Damage

**Figure 1:**
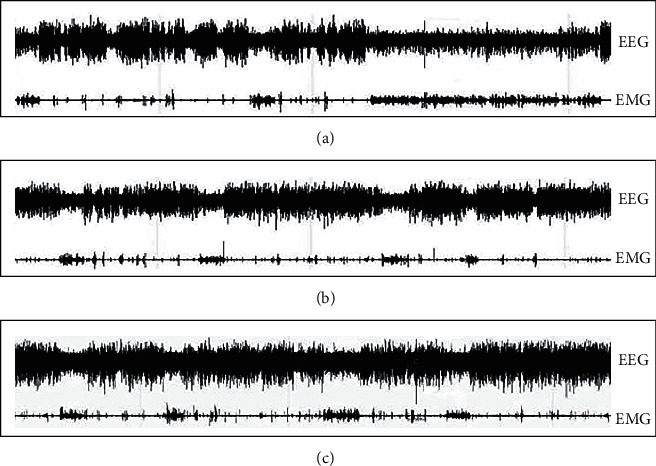
Typical examples of electroencephalogram and electromyogram damage. (a) The vestibular damage group. (b) The sham operation group. (c) The control group.

**Figure 2:**
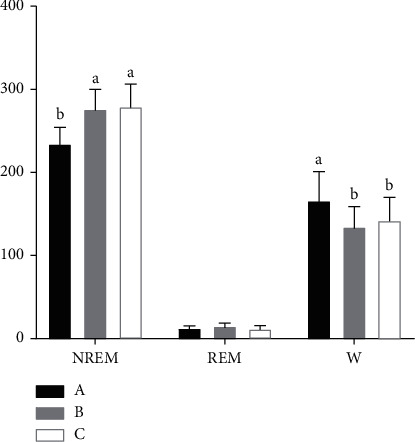
Effect of vestibular damage on the sleep-wakefulness cycle. A: the vestibular damage group; B: the sham operation group; C: the control group. NREM: nonrapid eye movement; REM: rapid eye movement; and W: wake period.

It can be seen from Figure 1 that compared with the control group, the vestibular damage group has a significant effect on the sleep of rats, while the sleep effect of rats in the sham operation group is not obvious. The waveform of high amplitude and low frequency in EEG represents sleep wave, the waveform of low amplitude and high frequency represents wake wave, and the EMG waveform indicates whether the animal has muscle activity. The high amplitude and slow wave of rats in the vestibular damage group are significantly reduced, and EMG shows that their myoelectric activity increases. The duration of NREM in the vestibular damage group decreases significantly compared with the control group (*P* < 0.05). The W time significantly increases compared with the control group (*P* < 0.05). Compared with the control group, the duration of REM in the sham operation group increases, but the difference is not statistically significant (*P* > 0.05) (Figure 2).

### 3.2. Effect of Vestibular Damage on the Level of Orexin mRNA and Autophagy in Hypothalamus

**Figure 3:**
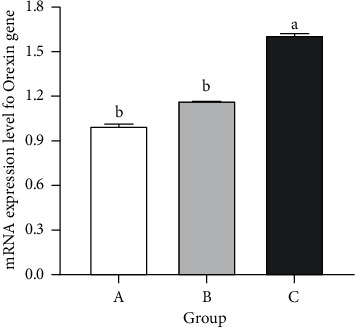
Effect of vestibular damage on the mRNA expression of orexin. A: the vestibular damage group; B: the sham operation group; and C: the control group. ^ab^ Values with different superscripts differ significantly (*P* < 0.05).

**Figure 4:**
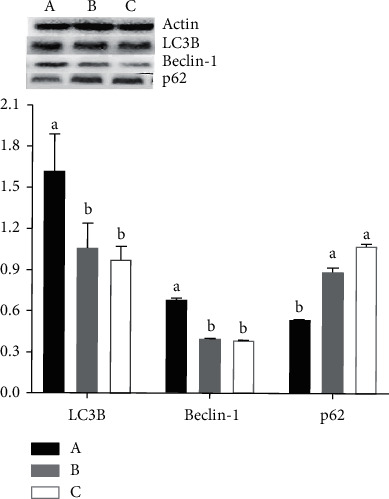
Effect of vestibular damage on the expression of autophagy protein (LC3B, Beclin-1, and p62). A: the vestibular damage group; B: the sham operation group; C: the control group. ^ab^Values with different superscripts differ significantly (*P* < 0.05).

Sion level of Orexin mRNA in the hypothalamus of rats in the vestibular damage group increases significantly compared with the control group and the sham operation group (*P* < 0.05), but there is no significant difference between the sham operation group and the blank group (*P* > 0.05) (Figure 3). Compared with the control group and sham operation group, the expression of autophagy microtubule related proteins LC3B and Beclin-1 in hypothalamus increase significantly (*P* < 0.05), while the protein expression level of p62 decreases significantly (*P* < 0.05). At the same time, the expression changes of three proteins in rat hypothalamus before the sham operation group and the control group have no significant statistical significance (*P* > 0.05) (Figure 4).

### 3.3. Orexin Effect of Orexin Intervention on the Viability of Hypothalamic Neurons

**Figure 5:**
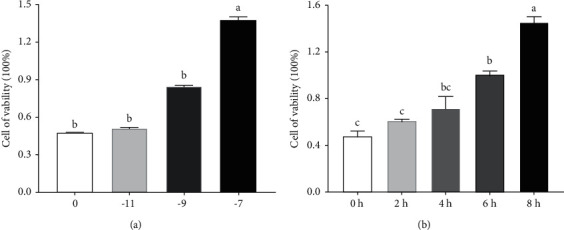
(a) Effects of different concentrations of orexin on hypothalamic neuronal cell viability. (b) Effects of 10^−7^ mol/L orexin stimulation on hypothalamus neuronal cell viability at different times. ^abc^Values with different superscripts differ significantly (*P* < 0.05).

Prepare orexin into four different concentrations of solutions to intervene in the same batch of cultured hypothalamic neuron cells for 2 h. It can be seen from Figure 5(a) that the cell viability increases in a concentration-dependent manner at the later stage of orexin intervention. Compared with the control group, the cell viability of 10–7 mol/L orexin intervention group increases significantly (*P* < 0.05).

### 3.4. Orexin Effect of Orexin on the Autophagy Level in Hypothalamic Neurons

**Figure 6:**
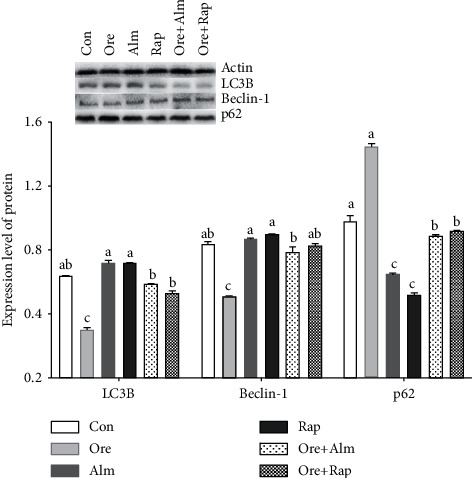
Expression levels of Beclin-1, LC3B, and P62 proteins in hypothalamus neurons induced by drugs. ^abc^Values with different superscripts differ significantly (*P* < 0.05).

In order to further quantitatively analyze the autophagy level of hypothalamic cells by orexin, 10^−7^ mol/L orexin is intervened for 6 hours as the orexin intervention group. After 6 groups of cells are collected, the expressions of autophagy related proteins p62, Beclin-1, and LC3B are detected. It can be seen from Figure 6 that after adding orexin, compared with the control group, the expression levels of Beclin-1 and LC3B proteins that are positively correlated with the autophagy activity decrease significantly (*P* < 0.05), and the expression level of p62 protein that is negatively correlated with the autophagy activity increases significantly (*P* < 0.05). In the two groups of cells in the orexin receptor inhibitor (Almorexant) group and the autophagy activator (Rapamycin) group, the expression of Beclin-1 and LC3B proteins increases significantly compared with the orexin intervention group (*P* < 0.05), and the expression level of the p62 protein decreases greatly compared with the control group (*P* < 0.05). In the cells of the orexin + receptor blocker group and orexin + autophagy activator group, it is observed that the inhibitory effect of orexin on the autophagy activity of cells can also be relieved to a certain extent. At the same time, the results of autophagy protein expression in the two groups are similar to those in the control group. However, compared with orexin group, the expression levels of Beclin-1 and LC3B are significantly different (*P* < 0.05), and the protein expression level of p62 significantly decreases (*P* < 0.05).

### 3.5. Orexin Regulates Autophagy through Akt Signal Pathway

**Figure 7:**
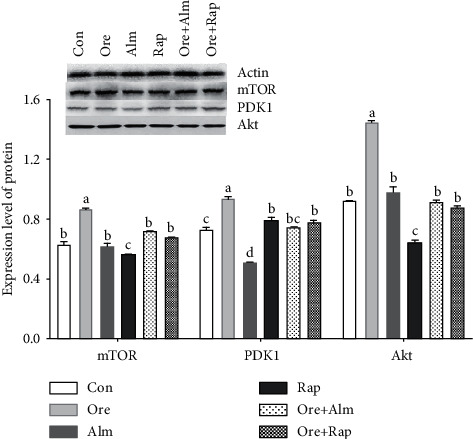
Expression levels of mTOR, PDK1, and Akt proteins in hypothalamus neurons under drug-induced conditions. ^abcd^Values with different superscripts differ significantly (*P* < 0.05).

It can be seen from Figure 7 that after orexin intervention, compared with the control group, the expression levels of mTOR, PDK1, and Akt protein increase significantly (*P* < 0.05). Compared with the orexin intervention group, the expression levels of mTOR, PDK1, and Akt proteins in cells in the Almorexant group, the Rapamycin group, the orexin + Almorexant group, and the orexin + rapamycin group decrease significantly (*P* < 0.05).

### 3.6. Inhibition of AKT/mTOR Signaling Pathway by EGCG Leads to Autophagy

**Figure 8:**
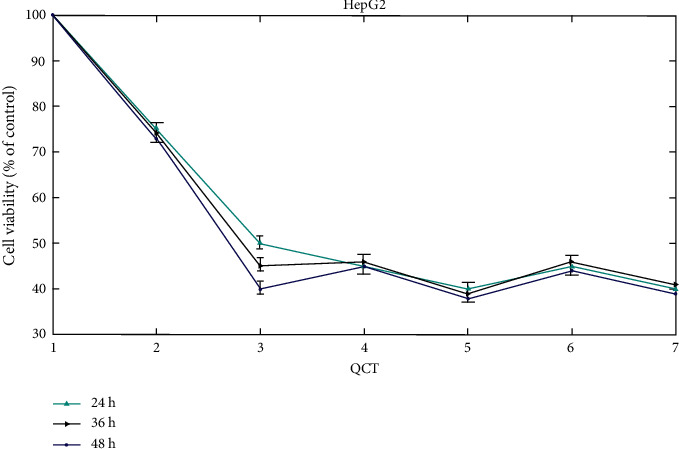
Viability of HepG2 cells.

Figure 8 shows the impacts of EGCG on the development of HCC tissues and regular hepatocytes are dose and time dependant. The CCK-8 assay was utilized to assess the vitality of HepG2 cells. The findings are represented as the averages and standard deviations (SD) of 3 independent tests. The value with a symbol is considerably varied from the control value *P* < 0.05.

**Figure 9:**
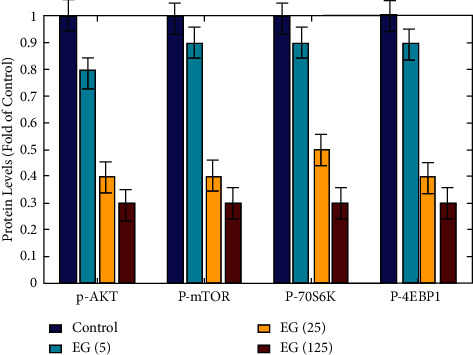
Protein-level analysis of P-Akt, P-mTOR, P-70S6K, and p-4EBP1.

**Figure 10:**
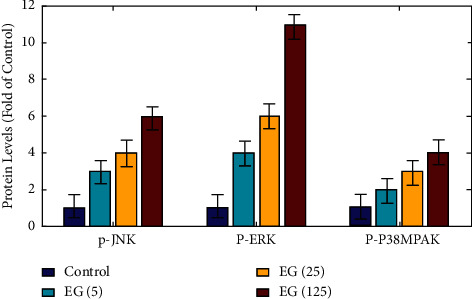
Protein-level analysis of P-JNK, P-ERK, and P-P38 MPAK.

Figure 9 shows the results of a densitometry examination of protein levels. Western blots are utilized to examine the impact of EGCG on the concentration of total and phosphorylated AKT, mTOR, p70S6K, and 4EBP1 proteins (*P* < 0.05).  Figure 10 shows JNK, ERK1/2, and p38MAPK, MAPK signaling pathway-associated target proteins, were also found to have high levels of expression. EGCG therapy elevated the protein content of phorylated JNK, ERK1/2, and p38MAPK in a dose-dependent way (*P* < 0.05), but had no impacts on the entire concentrations of these proteins.

## 4. Discussion

### 4.1. Effect of Vestibular Sleep-Wakefulness Cycle Changes in Rats with Vestibular Damage

Studies have found that the higher center of the vestibular system includes many subcortical and cortical structures that are related to the neural center of sleep [26–28]. Current studies believe that after the occurrence of sleep-wakefulness cycle stimulates the retina, the information is transmitted to the suprachiasmatic nucleus through the retinal hypothalamic pathway, and then a signal is sent to the pineal gland through the ganglion on the complex neural pathway to inhibit the secretion of melatonin so as to inhibit sleep and increase the wake state [29]. Pan et al. [30] also found that animals induced hyperactivity after bilateral vestibular injury. Therefore, the vestibule is a key area for sleep regulation. In order to clarify the effect of the vestibular system on the sleep-wakefulness cycle of rats, this experiment chooses to damage the vestibule by penetrating the tympanic membrane of rats' external auditory canals. The results show that the duration of NREM decreases significantly and the W time increases significantly. Therefore, vestibular damage affects the sleep-wakefulness cycle of the rats.

### 4.2. Orexin mRNA Effect of Vestibular Damage on the Level of Orexin mRNA and the Autophagy Level in Hypothalamus

Orexin has attracted much attention as a key neuropeptide for sleep-wakefulness control. Orexin and its receptor genes are knocked out or orexin neurons are denatured and deleted. The decrease of orexin level is listed as one of the diagnostic criteria of narcolepsy in animals and humans. Machaalani et al. [31] also found that the expression of orexin in hypothalamus of the rats with two vestibular damage models was significantly increased. The results of this study are the same. Orexin is closely related to vestibular system, which proves the correlation between vestibular system and sleep once again. Studies have found that orexin in HCT-116 human tumor cells can induce the autophagy activity through the ERK pathway [32]. There is yet no relevant literature report on whether orexin has an effect on the autophagy activity of hypothalamic neurons or whether it has the same effect as other peripheral cells in vitro so far.

### 4.3. Orexin Effect of Orexin on Autophagy of Hypothalamic Neurons

The autophagy process is completed by the corresponding proteins encoded by a series of autophagy-related genes (ATG), and LC3 protein is the homologue of yeast Atg8 in mammalian organism, which is considered to be the marker of autophagy [33]. p62 is a multidomain scaffold protein that binds to ubiquitinated modified proteins and transports them to autophagic vesicles for degradation. When autophagy occurs, p62 protein is continuously degraded. When autophagy is defective or its activity is weakened, p62 protein accumulates in the cytoplasm. Therefore, the level of p62 can be used as an indicator of the autophagy activity [34]. In addition, in the process of autophagy, the protein Beclin-1 is encoded by the BECN1 gene, which mainly binds to vps through the Bcl-2 functional domain to promote the occurrence of autophagy. When autophagy occurs, its expression increases, and vice versa, its content decreases. Like LC3 and p62, Beclin-l is one of the specific observation indexes of autophagy. The results of this study show that the activity of hypothalamic neuronal cells increases with the increase of orexin intervention concentration and intervention time; that is, orexin may have a certain effect on promoting the proliferation of hypothalamic neuronal cells. In order to further verify the intervention effect of orexin on hypothalamic neuronal cells, 10^−7^ mol/L of orexin with significant experimental results is selected for continuous intervention on cells for 6 hours. The expression of Beclin-1 and LC3B proteins, which are positively correlated with autophagy, is significantly reduced, while the accumulation of p62 increases significantly; in other words, orexin may promote cell activity and cell proliferation.

In this experiment, we found that the inhibitory effect of orexin on neuronal autophagy can be blocked by autophagy activator and orexin receptor blocker. Therefore, we speculate that orexin in neuronal cells may also achieve the function of inhibiting autophagy through the mTOR-dependent PI3K/Akt pathway. mTOR and PI3K/Akt are currently more familiar autophagy-related regulatory signal pathways, and it has been reported that Beclin-1 protein, a specific observation index of autophagy, is associated with PI3K/Akt/mTORC1 signal pathway. Further detection of the expression levels of mTOR, PI3K, and Akt proteins in the treated neuronal cells showed that orexin inhibited autophagy and then activated the mTOR-PI3K/Akt signal pathways to promote cell proliferation. Therefore, the sleep-wakefulness cycle disorder caused by tumor may regulate autophagy through hypothalamic orexin and affect tumor cell proliferation.

Hypocretin is two neuropeptides released mostly from the lateral hypothalamus that activate two specific receptors to exert a wide range of physiological effects. The researchers concentrate on the involvement of hypocretin signaling that is associated with amyloid brain layers and pain in rat, as well as tumor in cell lines and hypersomnia type. These findings point to hypocretin modulation having therapeutic ways in a variety of diseases, including narcolepsy and tumor. Catechin has been found to have anticancer properties linked to the stimulation of apoptosis and autophagy. The Akt/mTOR signaling pathway is a traditional and important autophagy negative regulatory route, as evidenced by new research. By regulating cellular proliferation, division, proliferation, apoptosis, and autophagy, MAPK signaling pathways display a part in the growth and advancement of tumor. JNK, ERK1/2, and p38MAPK are the 3 key members of standard MAPKs in mammalian cells. The JNK pathway is an autophagy regulatory route that is strongly positive. The ERK1/2 and p38 PAPK signaling pathways, on the other side, play multiple roles in autophagy, serving as both a positive and negative regulator. The findings demonstrate that EGCG activated autophagy by inhibiting Akt/mTOR and activating MAPK in in vitro functional assessments utilizing pathway-specific inhibitors or chemical activators. These findings support the theory that EGCG causes autophagy by regulating the Akt/mTOR pathway and activating the MAPK pathways. Although EGCG has a broad range of pharmacological properties, it has little pharmacological applicability due to its low and changeable bioavailability. To boost its bioavailability and in vivo efficiency, several techniques have been used, including prodrugs, nanocrystals, polymeric micelles, and microemulsions. Oral treatment of the pure state of EGCG at 61 mg/kg BW reduced the formation of SMMC7721 HCC cancers in vivo in our experiments. The more bioavailable form of EGCG is likely to be even more effective at inhibiting the growth of SMMC7721 cancers.

## 5. Conclusion

In conclusion, the expression of orexin in the hypothalamus of the rats increases significantly, and it inhibits autophagy of hypothalamic neurons and promotes cell viability and proliferation. In tumor-induced insomnia and sleep-wakefulness cycle disorder, orexin of the central nervous system may inhibit the autophagy of tumor cells through mTOR-dependent PI3K/Akt tumor signal pathways. Catechins can protect cardiovascular and cerebral vessels by activating autophagy.

## Figures and Tables

**Table 1:** Primer sequence.

Gene	Primer sequence (5′-3′)	Product size
GAPDH	F: TGAACGGGAAGCTCACTGG	307 bp
	R:TCCACCACCCTGTTGCTGTA	
Orexin	F:TCCTGCCGTCTCTACGAACT	133 bp
	R:GGTTACCGTTGGCCTGAAG	

## Data Availability

The datasets used during the current study are available from the corresponding author upon reasonable request.

## References

[1] Xu Z. P., Chen W. J., Li H. H. (2011). Study and treatment of insomnla. *Chinese Jourud of Clinical Rehabilitation*.

[2] Zhou J., Liu S. X. (2009). Research progress in the effect of sleep disor-der on immune-related response and diseases. *E-Journal of Translational Medicine*.

[3] Wiest G. (2015). The origins of vestibular science. *Annals of the New York Academy of Sciences*.

[4] Normand H., Etard O., Denise P. (1997). Otolithic and tonic neck receptors control of limb blood flow in humans. *Journal of Applied Physiology (1985)*.

[5] Yates B. J., Billig I., Cotter L. A., Mori R. L., Card J. P. (2010). Role of the vestibular system in regulating respiratory muscle activity during movement. *Clinical and Experimental Pharmacology and Physiology*.

[6] Fuller P. M., Jones T. A., Jones S. M., Fuller C. A. (2002). Neurovestibular modulation of circadian and homeostatic regulation: vestibulohypothalamic connection?. *Proceedings of the National Academy of Sciences of the United States of America*.

[7] Highstein S. M. (2004). *Anatomy and Physiology of the Central and Peripheral Vestibular System: Overview*.

[8] Yates B. J. (1996). Vestibular influences on the autonomic nervous system. *Annals of the New York Academy of Sciences*.

[9] Balaban C. D., Yates B. J. (2004). Vestibuloautonomic interactions: a teleologic perspective. *Springer Handbook Auditory Research*.

[10] de Lecea T. S., Gao X. B., Kilduff C. (1998). The hypocretins: hypothalamus-specific peptides with neuroexcitatory activity. *Proceedings of the National Academy of Sciences*.

[11] Sakurai T., Amemiya A., Ishii M. (1998). Orexins and orexin receptors: a family of hypothalamic neuropeptides and G protein-coupled receptors that regulate feeding behavior. *Cell*.

[12] Marcus J. N., Aschkenasi C. J., Lee C. E. (2001). Differential expression of orexin receptors 1 and 2 in the rat brain. *Journal of Comparative Neurology*.

[13] Laburthe M., Voisin T. (2012). The orexin receptor OX_1_R in colon cancer: a promising therapeutic target and a new paradigm in G protein-coupled receptor signalling through ITIMs. *British Journal of Pharmacology*.

[14] The Pharmaceutical Journal Group (2007). Orexin antagonist shows promise as insomnia treatment. *The Pharmaceutical Journal*.

[15] Wang Z., Liu S., Kakizaki M. (2014). Orexin/hypocretin activates mTOR complex 1 (mTORC1) via an erk/akt-independent and calcium-stimulated lysosome v-ATPase pathway. *Journal of Biological Chemistry*.

[16] Ju S. J., Zhao Y., Chang X., Guo L. (2014). Orexin A protects cells from apoptosis by regulating FoxO1 and mTORC1 through the OX1R/PI3K/AKT signaling pathway in hepatocytes. *International Journal of Molecular Medicine*.

[17] Maday S., Wallace K. E., Holzbaur E. L. F. (2012). Autophagosomes initiate distally and mature during transport toward the cell soma in primary neurons. *Journal of Cell Biology*.

[18] Berger A. M. (2009). Update on the state of the science: sleep-wakefulness disturbances in adult patients with cancer. *Oncology Nursing Forum*.

[19] Savard J., Simard S., Ivers H., Morin C. M. (2005). Randomized study on the efficacy of cognitive-behavioral therapy for insomnia secondary to breast cancer, part I: sleep and psychological effects. *Journal of Clinical Oncology Official Journal of the American Society of Clinical Oncology*.

[20] Sun Y., Liu J. H., Jin L. (2010). Over-expression of the beclin1 gene upregulates chemosensitivity to anti-cancer drugs by enhancing therapy-induced apoptosis in cervix squamous carcinoma caski cells. *Cancer Letters*.

[21] Sun Y., Liu J. H., Pan L. (2011). Modulatory effects of Beclin 1 on expression of angiopoietin and Tie-2 receptor in human cervical cancer cells. *Asian Pacific Journal of Cancer Prevention*.

[22] Lee S. J., Kim H. P., Jin Y., Choi A. M., Ryter S. W. (2011). Beclin 1 deficiency is associated with increased hypoxia-induced angiogenesis. *Autophagy*.

[23] Wu C. L., Zhang S. M., Lin L. (2018). BECN1-knockout impairs tumor growth, migration and invasion by suppressing the cell cycle and partially suppressing the epithelial-mesenchymal transition of human triple-negative breast cancer cells. *International Journal of Oncology*.

[24] Aita V. M., Liang X. H., Murty V. V. (1999). Cloning and genomic organization of beclin 1, a candidate tumor suppressor gene on chromosome 17q21. *Genomics*.

[25] Nishikawa M., Miyake H., Liu B., Fujisawa M. (2015). Expression pattern of autophagy-related markers in non-metastatic clear cell renal cell carcinoma: association with disease recurrence following radical nephrectomy. *Journal of Cancer Research and Clinical Oncology*.

[26] Huo Y., Cai H., Teplova I. (2013). Autophagy opposes p53-mediated tumor barrier to facilitate tumorigenesis in a model of PALB2-associated hereditary breast cancer. *Cancer Discovery*.

[27] Lopez C., Blanke O. (2011). The thalamocortical vestibular system in animals and humans. *Brain Research Reviews*.

[28] Dieterich M., Brandt T. (2015). The bilateral central vestibular system: its pathways, functions, and disorders. *Annals of the New York Academy of Sciences*.

[29] Saper C. B., Chou T. C., Scammell T. E. (2001). The sleep switch: hypothalamic control of sleep and wakefulness. *Trends in Neurosciences*.

[30] Pan L., Qi R., Wang J., Zhou W., Liu J., Cai Y. (2016). Evidence for a role of orexin/hypocretin system in vestibular lesion-induced locomotor abnormalities in rats. *Frontiers in Neuroscience*.

[31] Machaalani R., Hunt N. J., Waters K. A. (2013). Effects of changes in energy homeostasis and exposure of noxious insults on the expression of orexin (hypocretin) and its receptors in the brain. *Brain Research*.

[32] Wen J., Zhao Y. Y. (2016). Orexin A induces autophagy in HCT-116 human colon cancer cells through the ERK signaling pathway. *International Journal of Molecular Medicine*.

[33] Dong Y. (2010). Autophagy: definition, molecular machinery, and potential role in myocardial ischemia repenfusion injury. *Journal of Cardiovascular Pharmacology and Therapeutics*.

[34] Bordi M., Berg M. J., Mohan P. S. (2016). Autophagy flux in CA1 neurons of Alzheimer hippocampus: increased induction overburdens failing lysosomes to propel neuritic dystrophy. *Autophagy*.

